# Neuroanatomical correlates of psychiatric manifestations in Wilson disease: the role of age

**DOI:** 10.3389/fpsyt.2026.1960346

**Published:** 2026-09-18

**Authors:** Anqin Wang, Chunsheng Xu, Danqing Liu, Man Jiang, Chuanfu Li

**Affiliations:** 1 Imaging Center, The First Affiliated Hospital of Anhui University of Chinese Medicine, Hefei, Anhui, China; 2 Department of Neurology, The First People’s Hospital of Mengcheng County, Mengcheng County, Anhui, China

**Keywords:** age, gray matter, psychiatric, voxel-based morphometry, Wilson’s disease

## Abstract

**Objective:**

To investigate gray matter volume (GMV) differences related to psychiatric manifestations in patients with Wilson disease (WD) and to examine the effect of age.

**Methods:**

In 102 patients with WD, 3.0T T1-weighted MRI scans were analyzed using voxel-based morphometry. Patients were divided into younger (≤30 years) and older (>30 years) groups and were further grouped according to psychiatric manifestations. Patients with a UWDRS psychiatric subscale (UWDRS-PSY) score of 0 were classified as having no psychiatric manifestations, and those with a score >0 were classified as having psychiatric manifestations. Two-way ANCOVA was performed with total intracranial volume, sex, and education as covariates. Cluster-level family-wise error (FWE) correction was applied. Mean GMV values were extracted from significant caudate clusters and correlated with UWDRS-PSY scores as exploratory analyses.

**Results:**

Significant interactions between age group and psychiatric manifestations were found in the left insula/Rolandic operculum and right inferior temporal gyrus. Lower GMV in these regions was mainly found in younger patients with psychiatric manifestations. A main effect of psychiatric manifestations was found in the bilateral caudate nuclei, with lower GMV in patients with psychiatric manifestations. Lower caudate GMV was also associated with higher UWDRS-PSY scores.

**Conclusions:**

Psychiatric manifestations in WD are associated with distinct gray matter patterns that vary with age, while bilateral caudate involvement may represent a more consistent structural correlate. These findings highlight the potential importance of considering age when examining the neuroanatomical correlates of psychiatric manifestations in WD.

## 1 Introduction

Wilson disease (WD) is an autosomal recessive disorder of copper metabolism characterized by progressive multisystem involvement, most commonly affecting the central nervous system and liver (1). Although neurological symptoms (primarily extrapyramidal symptoms) have been extensively documented, psychiatric manifestations are frequently underestimated. A growing body of research indicates that psychiatric comorbidities in WD exert varying degrees of impact on social functioning and health-related quality of life. Early recognition of these challenges is difficult, leading to reduced treatment adherence and complexities in long-term disease management (2–4).

From a neuropathological perspective, WD primarily affects the basal ganglia and associated cortico-subcortical circuits within the nervous system. These circuits govern cognitive control, reward evaluation, and behavioral regulation (5). These networks play a central role in emotional regulation and executive function. Kalit scholars have demonstrated that alterations in the structure of the cortico-striatal system can trigger psychiatric manifestations (6). Despite theoretical underpinnings, neuroimaging evidence revealing brain structural alterations associated with psychiatric manifestations in Wilson’s disease remains scarce and methodologically diverse. Existing studies predominantly focus on motor dysfunction or global neuroanatomical abnormalities, with psychiatric manifestations often treated as secondary outcomes rather than assessed as primary features via clinical scales (5, 7, 8).

Age may also influence the relationship between psychiatric manifestations and brain structure. Gray matter development and neural network refinement persist into early adulthood, implying that brains at different ages exhibit varying degrees of structural vulnerability to injury (9, 10).

Therefore, the same disease-related burden may be associated with different brain structural patterns at different ages. We hypothesized that younger patients with WD may show more evident gray matter differences related to psychiatric manifestations.

This study employed voxel-based morphometry (VBM) to investigate the association between psychiatric manifestations and gray matter volume in patients with WD. We examined the main effect of psychiatric manifestations and the interaction between age group and psychiatric-manifestation status while controlling for relevant demographic covariates. We further explored the association between regional gray matter volume and the severity of psychiatric manifestations assessed by the UWDRS-PSY. The aim was to better understand the age-related neuroanatomical characteristics of psychiatric manifestations in WD.

## 2 Methods

### 2.1 Participants and study design

This study protocol has been reviewed and approved by the Ethics Committee of the First Affiliated Hospital of Anhui University of Chinese Medicine (2021AH-60). All subjects signed written informed consent forms prior to enrollment. A total of 102 patients with Wilson’s disease were recruited from the Department of Neurology at the First Affiliated Hospital of Anhui University of Chinese Medicine between 2021 and 2024. The cohort comprised 63 males and 39 females, ranging in age from 12 to 56 years, with a mean age of (27.69 ± 8.58) years. All subjects were right-handed. All cases were diagnosed according to the Leipzig diagnostic criteria. Exclusion criteria included: (1) patients with concomitant neurological or psychiatric disorders unrelated to Wilson disease, and (2) patients with contraindications for MRI examination.

### 2.2 Clinical and biochemical assessments

Clinical and biochemical data were collected for all participants, including age, sex, serum ceruloplasmin (SC), 24-hour urinary copper excretion (24-h UC), educational level, presence of Kayser–Fleischer (K–F) rings, and family history. Disease severity was assessed using the Chinese version of the full 55-item Unified Wilson Disease Rating Scale (UWDRS), which has undergone reliability and validity evaluation in a Chinese population. The full UWDRS comprises three subscales: the neurological subscale (items 1–27; score range, 0–208), the hepatic subscale (items 28–36; score range, 0–36), and the psychiatric subscale (items 37–55; score range, 0–76), yielding a maximum total score of 320 (11, 12). The total UWDRS score ranges from 0 to 320, with higher scores indicating more severe clinical manifestations. Each participant was independently assessed by two attending physicians using the UWDRS. The two raters performed the assessments independently according to the predefined item-specific scoring criteria of the scale, and the mean of the two ratings was used as the final score for subsequent analyses.

### 2.3 Image acquisition

All MRI data were acquired using a 3.0T scanner (Discovery MR750, GE Healthcare, Milwaukee, Wisconsin, USA) with an 8-channel head radiofrequency coil. Whole-brain structural scans were performed in planes parallel to the anterior-posterior commissure (AC-PC). Foam padding secured the head during scanning to minimize motion, and earplugs were provided to reduce scanning noise. Participants were instructed to remain awake and still with eyes closed, avoiding conscious thought activity.High-resolution three-dimensional T1-weighted structural images were obtained using a sagittal sequence (TR = 8.2 ms, TE = 3.2 ms, FA = 12°, FOV = 256 mm × 256 mm, matrix = 256 × 256, slice thickness = 1 mm, interslice gap = 0 mm, 166 slices), after which all images were visually inspected; datasets showing evident artifacts or substantial head motion were excluded from further analysis.

### 2.4 VBM pre-processing

Three-dimensional T1-weighted structural images were preprocessed using the Computational Anatomy Toolbox (CAT12; Structural Brain Mapping Group, Jena University Hospital, Germany) implemented in SPM12 (Wellcome Centre for Human Neuroimaging, London, UK) on the MATLAB platform (MathWorks, Natick, MA, USA). Preprocessing was conducted according to the standard CAT12 workflow. Raw images were first converted from DICOM to NIfTI format using the dcm2nii utility in MRIcron. CAT12 was then used for bias-field correction, denoising, skull stripping, and automated segmentation into gray matter (GM), white matter (WM), and cerebrospinal fluid (CSF). The GM and WM maps were spatially normalized to the Montreal Neurological Institute (MNI) template using the DARTEL algorithm and resampled to a voxel size of 1.5 mm³. After spatial normalization, the normalized GM maps were modulated using Jacobian determinants to preserve regional GM volume. Quality control was performed at two levels. First, the normalized images were visually checked for artifacts and misregistration. Second, the correlation coefficient between each normalized GM image and the standard template was calculated to assess image similarity. Images with a correlation coefficient ≤0.85 were excluded. A total of 102 participants passed quality control and were included in the final VBM analysis. Finally, the modulated and normalized GM images were smoothed using an 8-mm full width at half maximum (FWHM) Gaussian kernel to reduce individual anatomical differences and improve the stability of voxel-wise analysis.

### 2.5 Statistical analyses

Continuous variables are presented as mean ± standard deviation (SD) or median (interquartile range [IQR]), as appropriate. Normally distributed variables were compared using the independent-samples t test, and nonnormally distributed variables were compared using the Mann–Whitney U test. Categorical variables are presented as n (%) and were compared using the Pearson chi-square test or Fisher’s exact test, as appropriate. Missing values were not imputed. All tests were two-sided, and P<0.05 was considered statistically significant. Psychiatric manifestations were defined according to the psychiatric subscale of the UWDRS-PSY.

. Patients with a UWDRS-PSY score of 0 were classified as having no psychiatric manifestations, whereas those with a score >0 were classified as having psychiatric manifestations. The psychiatric subscale covers heterogeneous manifestations, including sleep disturbance, mood and anxiety symptoms, cognitive and behavioral changes, and psychotic-like manifestations such as persecutory ideation and auditory or visual hallucinations.

Participants were divided into a younger group (≤30 years) and an older group (>30 years). According to age and psychiatric-manifestation status, the participants were further divided into four subgroups: younger patients without psychiatric manifestations, younger patients with psychiatric manifestations, older patients without psychiatric manifestations, and older patients with psychiatric manifestations. The 30-year cutoff was selected because brain structural maturation continues into early adulthood (13). To test the stability of this age cutoff, sensitivity analyses were performed using cutoffs of 28 and 32 years and by excluding participants within ±2 years of the 30-year cutoff. The direction of the results remained consistent across these analyses.

Voxel-wise GMV was analyzed using a two-way analysis of covariance (ANCOVA) to examine the main effect of psychiatric manifestations and the interaction between age group and psychiatric-manifestation status. Total intracranial volume (TIV), sex, and educational level were included as covariates. Statistical maps were first thresholded at voxel-wise p < 0.001, and clusters with cluster-level FWE-corrected p < 0.05 were considered statistically significant. For the exploratory correlation analysis, brain regions showing a significant main effect of psychiatric manifestations were converted into binary masks. For each participant, the mean GMV within each significant cluster was extracted from the preprocessed GM images. Pearson correlation analysis was then used to examine the association between the extracted mean GMV and UWDRS-PSY scores.

## 3 Results

### 3.1 Participant characteristics

Among the 102 patients with WD, 39 had a UWDRS-PSY score of 0 and 63 had a score >0. The four age × psychiatric-manifestation subgroups included 27 younger patients without psychiatric manifestations, 38 younger patients with psychiatric manifestations, 12 older patients without psychiatric manifestations, and 25 older patients with psychiatric manifestations. Participant characteristics are shown in **Table 1**. Age, sex, age-group distribution, educational level, K–F ring status, and 24-hour urinary copper did not differ significantly between the two psychiatric-manifestation groups (all P>0.05). Patients with psychiatric manifestations had a higher proportion of positive family history (25.4% vs 7.7%, P = 0.035) and lower serum ceruloplasmin levels (P = 0.020) (**Table 1**).

**Table 1:** Demographic and clinical characteristics according to psychiatric manifestations.

Characteristics	Total (n=102)	UWDRS-PSY = 0 (n=39)	UWDRS-PSY > 0 (n=63)	P value
Age, years	27.69 ± 8.58	27.44 ± 7.96	27.84 ± 8.99	0.818
Sex, male, n (%)	63 (61.8%)	23 (59.0%)	40 (63.5%)	0.648
Age group, n (%)				0.363
≤30 years	65 (63.7%)	27 (69.2%)	38 (60.3%)	
>30 years	37 (36.3%)	12 (30.8%)	25 (39.7%)	
Educational level, n (%)				0.920
Primary school	9 (8.8%)	4 (10.3%)	5 (7.9%)	
Junior middle school	33 (32.4%)	13 (33.3%)	20 (31.7%)	
Senior secondary/vocational	22 (21.6%)	9 (23.1%)	13 (20.6%)	
College or above	38 (37.3%)	13 (33.3%)	25 (39.7%)	
Positive family history, n (%)	19 (18.6%)	3 (7.7%)	16 (25.4%)	0.035
K–F ring positive, n (%)	92 (90.2%)	37 (94.9%)	55 (87.3%)	0.310
24-h urinary copper	767.08 (433.67–1108.11)	752.52 (515.07–1104.33)	767.08 (417.58–1107.82)	0.724
Serum ceruloplasmin	0.0405 (0.0240–0.0517)	0.0445 (0.0365–0.0558)	0.0330 (0.0187–0.0488)	0.020

Bold values indicate statistically significant differences (p < 0.05).

### 3.2 Age × psychiatric status interaction effects

A two-way analysis of covariance (ANCOVA) was conducted to examine whether the association between psychiatric-manifestation status and gray matter volume was moderated by age. The results revealed statistically significant age × psychiatric-manifestation status interactions in two brain regions: the left insula/Rolandic_Oper_gyrus and the right inferior temporal gyrus (**Table 2**). In the left insula/Rolandic area, the interaction term was significant (F = 5.97, p=0.016, η_p_²=0.059), indicating that mental state-related volume differences varied across age groups. Subsequent age-stratified analysis revealed: in the younger group, patients with psychiatric manifestations exhibited significantly lower gray matter volume than those without psychiatric manifestations (p<0.01); whereas no statistically significant intergroup differences were observed in the older group (**Figure 1**). The right inferior temporal gyrus exhibited a similar modulation pattern, with the age × psychiatric-manifestation status interaction also reaching statistical significance (F = 8.86, p=0.004, η_p_²=0.084). In this region, the lower gray matter volume in the psychiatric manifestation subgroup was also significant only in the younger cohort (p < 0.01), while the difference between psychiatric manifestation and non-psychiatric manifestations in older participants was indistinguishable from zero (**Figure 2**).

**Table 2:** Brain regions showing significant age × psychiatric-manifestation interaction effects in the two-way ANCOVA.

Brain region	Peak MNI coordinates			Cluster size (voxels)	P value	F value	η_p_²
	X	Y	Z				
Temporal_Inf_R	57	-15	-31.5	876	0.004	8.860	0.084
Insula/Rolandic_Oper_L	-45	-14	16	555	0.016	5.971	0.059

**Figure 1:**
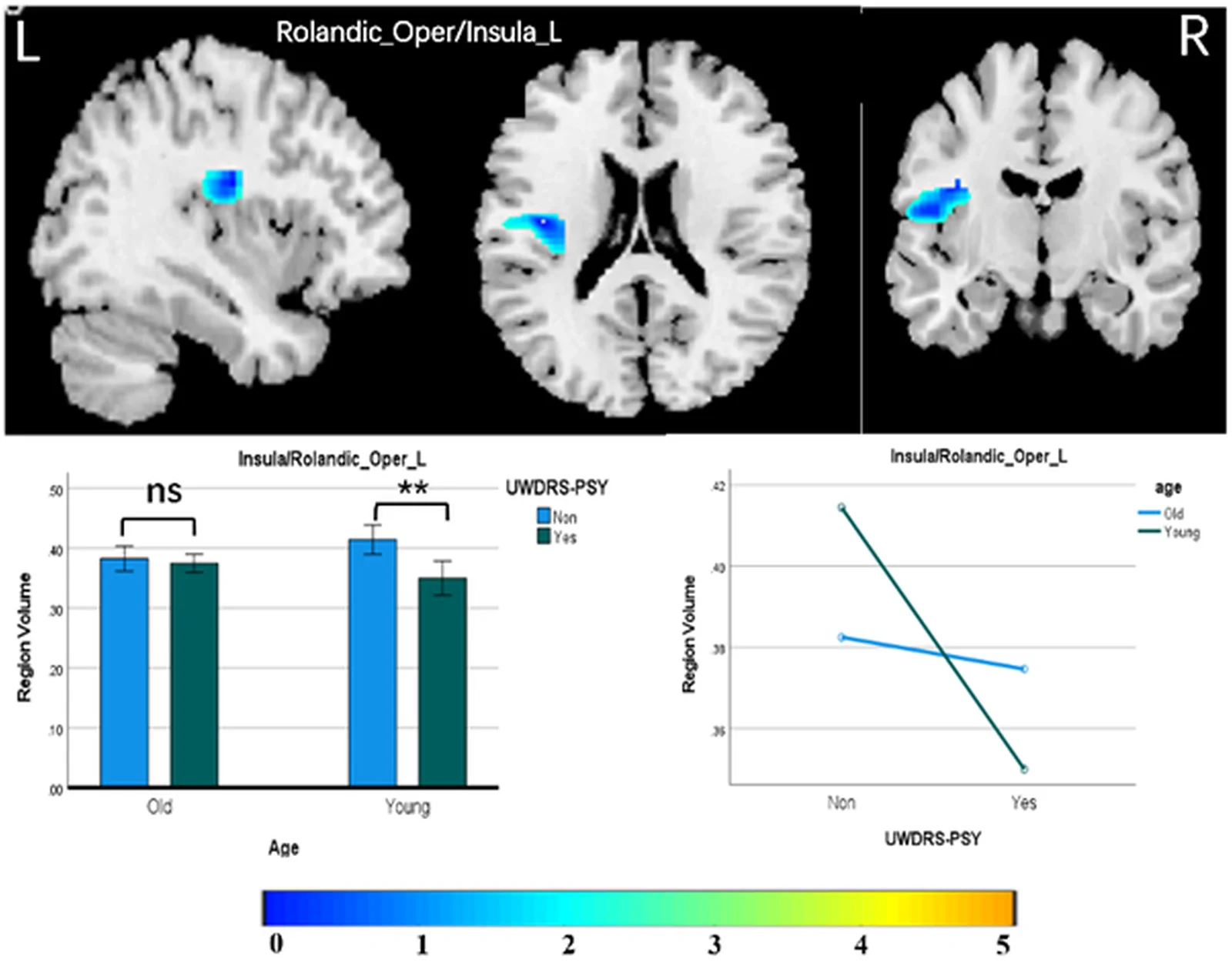
The upper panels display the significant gray matter cluster identified in the two-way ANCOVA (voxel-wise p < 0.001, cluster-level FWE p < 0.05). The lower right panel shows the bar plot of group gray matter volumes, and the lower left panel presents the interaction line plot illustrating the age and psychiatric-status grouping. The bottom panel provides the color bar representing statistical intensity. ** indicates p < 0.01; ns indicates not significant.

**Figure 2:**
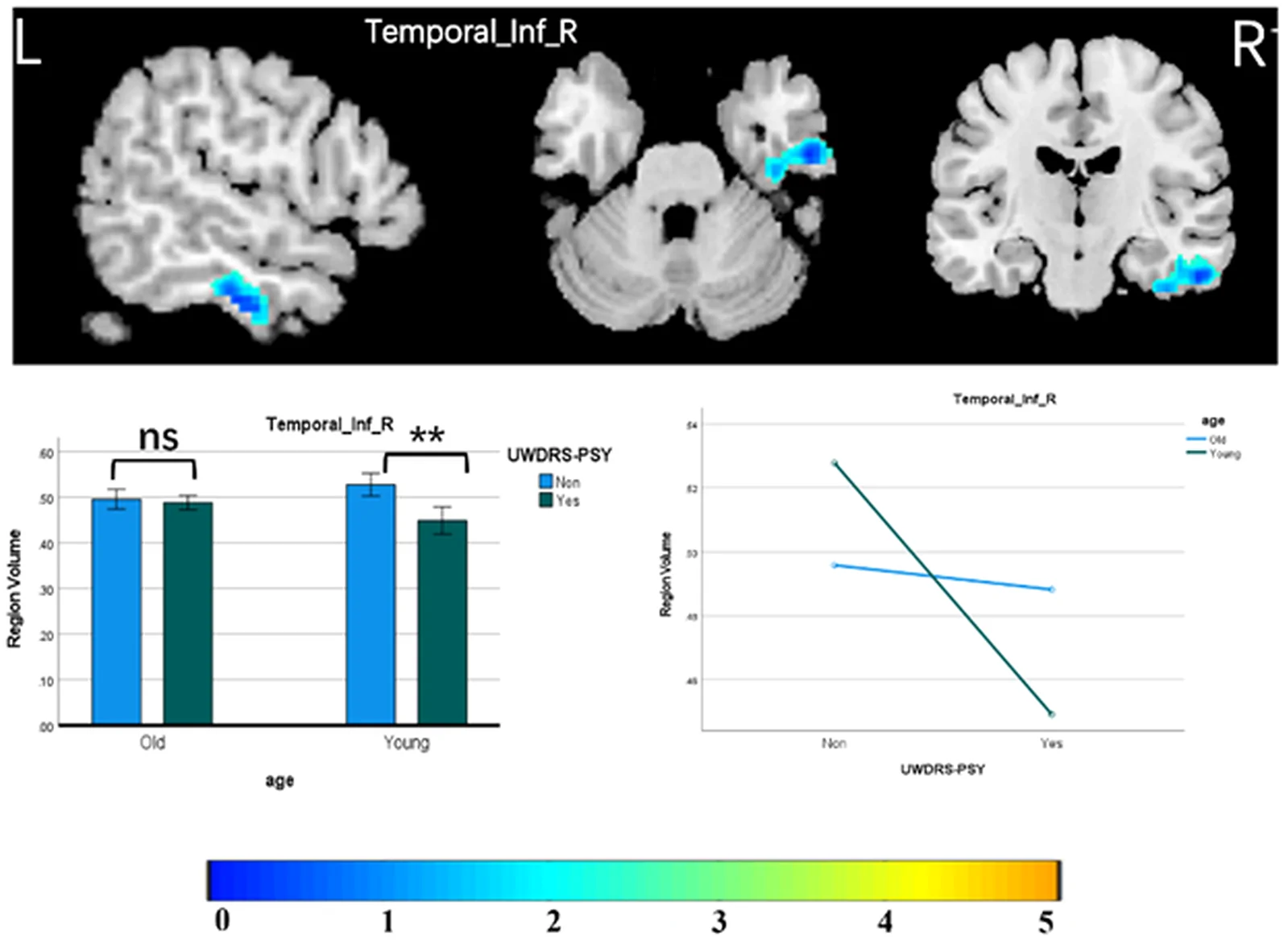
The upper panels display the significant gray matter cluster in the right inferior temporal gyrus identified in the two-way ANCOVA (voxel-wise p < 0.001, cluster-level FWE p < 0.05). The lower right panel shows the bar plot of group gray matter volumes, and the lower left panel presents the interaction line plot illustrating the age and psychiatric-status grouping. The bottom panel provides the color bar representing statistical intensity.

### 3.3 Main effect of psychiatric manifestations

In the main-effect analysis of psychiatric manifestations, VBM revealed significant gray matter volume (GMV) reductions in the bilateral caudate nuclei (**Table 3**). Compared with WD patients without psychiatric manifestations, those with psychiatric manifestations showed a marked decrease in GMV in the right caudate nucleus (p = 0.002, Cohen’s d = 0.644) **Figure 3A**. In addition, GMV in the right caudate was significantly negatively correlated with UWDRS-PSY scores (r = −0.36, p < 0.001). Similarly, patients with psychiatric manifestations exhibited significantly lower GMV in the left caudate nucleus than those without psychiatric manifestations (p = 0.001, Cohen’s d = 0.675), and left caudate GMV was also negatively correlated with UWDRS-PSY scores (r = −0.34, p < 0.001) **Figure 3B**, indicating that greater psychiatric manifestations severity was associated with smaller caudate GMV.

**Table 3:** Gray matter volume differences in the bilateral caudate nuclei of WD patients under the main effect of psychiatric factors.

Brain region	Peak MNI coordinates			Cluster size (voxels)	Mean (SD)		P value	T value	Cohen’s d
	X	Y	Z		Non-psy	Psy			
Caudate_R	6	18	6	673	0.36 (0.1)	0.30 (0.09)	0.002	3.180	0.644
Caudate_L	-10.5	-24	4.5	670	0.29 (0.09)	0.24 (0.07)	0.001	3.333	0.675

**Figure 3:**
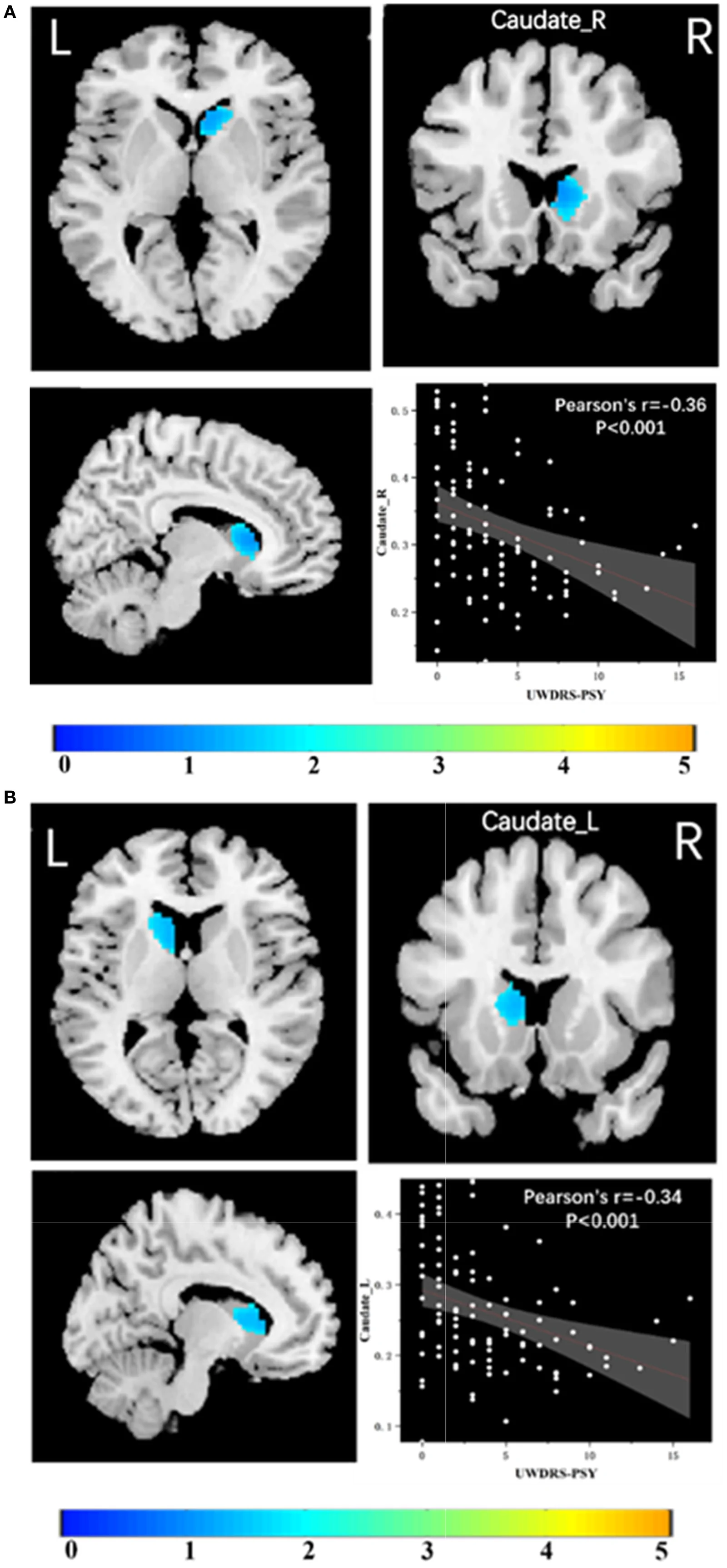
**(A)** The upper panels and the lower left panel show the brain region with a significant main effect of psychiatric factors identified by the two-way ANCOVA (right caudate nucleus). The lower right panel presents the scatter plot with linear regression illustrating the association between gray matter volume in the right caudate nucleus and psychiatric manifestations scores (UWDRS-PSY). The color bar at the bottom indicates statistical intensity. **(B)** The upper panels and the lower left panel show the brain region with a significant main effect of psychiatric factors identified by the two-way ANCOVA (left caudate nucleus). The lower right panel presents the scatter plot with linear regression illustrating the association between gray matter volume in the left caudate nucleus and psychiatric manifestations scores (UWDRS-PSY). The color bar at the bottom indicates statistical intensity.

## 4 Discussion

In this study, we used two-way ANCOVA and VBM to investigate the structural correlates of psychiatric manifestations in WD, while controlling for key confounders (TIV, sex, and education) and applying cluster-level FWE correction. Our main findings are as follows. First, an age-by-psychiatric manifestations interaction was observed in the left insula/Rolandic operculum and the right inferior temporal gyrus, with psychiatric manifestations primarily associated with reduced GMV in the younger group, whereas no significant differences were found in older patients. Second, psychiatric manifestations showed a robust main effect on bilateral caudate GMV, with patients exhibiting significantly lower GMV in both the right and left caudate nuclei, and caudate GMV negatively correlating with the severity of psychiatric manifestations (UWDRS-PSY).

### 4.1 Interaction in insular and temporal regions

We first observed a significant age × psychiatric status interaction in the left insula/Rolandic operculum and the right inferior temporal gyrus, with psychiatric manifestations being associated with reduced GMV predominantly in younger patients but not in older patients. This pattern suggests that the neuroanatomical signature of psychiatric involvement in WD may be age-dependent, potentially reflecting a window of heightened vulnerability during late adolescence/early adulthood when large-scale brain organization is still undergoing non-linear reconfiguration and has not fully stabilized (9, 10). Mousley’s life-span connectome research revealed that the developmental trajectory of brain network topology does not conclude entirely by the early twenties; this developmental process extends to approximately age 32, with a distinct developmental inflection point occurring around this age (14). Therefore, we used 30 years as the age cutoff in the present study. Furthermore, during the transition from adolescence to early adulthood, individual variations in brain structural changes exist and become more pronounced during this transitional phase. These inter-individual differences may alter the extent to which disease burden influences brain structure. In other words, the same disease burden may not necessarily manifest as equally observable neuroanatomical differences across different individuals (15, 16).

The insula/operculum is involved in emotional processing, salience detection, and behavioral regulation (17). The anterior insula is a core region of the salience network and is involved in detecting important internal and external information and coordinating cognitive and emotional processing (18, 19). The right inferior temporal gyrus is involved in higher-order visual, semantic, and socio-emotional processing. Structural differences in this region may therefore be related to cognitive and emotional manifestations in WD (20–23).

The absence of significant differences in the older group should be interpreted cautiously. Greater clinical and structural heterogeneity in older patients may have reduced the ability to detect between-group differences. In addition, the relatively small number of older patients without psychiatric manifestations may have limited statistical power. Therefore, the present findings do not provide direct evidence of compensatory changes in older patients. These findings suggest that younger patients with WD may require particular attention to psychiatric manifestations during clinical assessment.

### 4.2 Caudate structure and psychiatric manifestations

Patients with psychiatric manifestations showed lower GMV in the bilateral caudate nuclei. In present analyses, lower caudate GMV was also associated with higher UWDRS-PSY scores. These findings suggest that the caudate may be related to psychiatric manifestations in WD. From a neuroanatomical perspective, the caudate nucleus is an important component of corticostriatal circuits and has extensive connections with the prefrontal cortex and limbic regions. It is involved in cognitive control, reward-guided behavior, action selection, motivation, and behavioral regulation (24). Structural abnormalities of the caudate have also been reported in several psychiatric conditions, supporting its general role in emotional, cognitive, and behavioral regulation (25). However, psychiatric manifestations in WD are heterogeneous and should not be directly equated with primary psychiatric disorders.

Basal ganglia involvement is a well-recognized feature of WD. Previous structural MRI studies have reported abnormalities involving the caudate and other subcortical nuclei in patients with WD (5, 7). These findings suggest that the caudate is one of the brain regions susceptible to WD-related brain injury. In the present study, patients with psychiatric manifestations showed lower bilateral caudate GMV, further suggesting that caudate structural differences may be related to the psychiatric manifestations of WD.

Previous studies have also suggested an association between basal ganglia involvement and neuropsychiatric manifestations in WD. Kalita et al. reported relationships between MRI abnormalities and neuropsychiatric manifestations in patients with WD (6). ATP7B dysfunction and abnormal copper accumulation may contribute to oxidative stress, mitochondrial dysfunction, altered neurotransmission, and neuroinflammatory changes, which may affect vulnerable subcortical regions such as the basal ganglia. In addition, because the caudate is an important component of corticostriatal circuits involved in cognitive, emotional, and behavioral regulation, structural changes in this region may be related to psychiatric manifestations in WD. However, the present cross-sectional study cannot determine whether these mechanisms directly account for the observed caudate GMV differences or clarify whether the structural changes precede, accompany, or follow the development of psychiatric manifestations.

### 4.3 Corticostriatal circuits and psychiatric manifestations

Within the cortico-striato-thalamo-cortical (CSTC) circuit, the caudate nucleus plays an important role in cognitive control, action selection, and behavioral regulation. Through its connections with the prefrontal cortex, thalamus, and other subcortical regions, the caudate participates in the regulation of cognitive, emotional, and behavioral processes (26). Therefore, the lower caudate GMV observed in patients with psychiatric manifestations may reflect structural involvement of brain systems related to these functions.

Previous neuroimaging studies provide some support for the involvement of corticostriatal networks in WD. Tinaz et al. reported structural abnormalities in the striatum and thalamus and reduced functional connectivity between frontostriatal regions in patients with neurological WD. These findings suggest that WD-related brain injury may involve both the structure and functional organization of corticostriatal systems (26). In addition, Wang et al. found widespread alterations in resting-state brain networks in patients with WD, and some functional connectivity changes were associated with UWDRS psychiatric scores (27). These findings indicate that psychiatric manifestations in WD may be related not only to changes in individual brain regions but also to alterations in broader brain networks.

The age-related findings in the present study may also provide additional information. Psychiatric-manifestation-related GMV differences in the insula/Rolandic operculum and inferior temporal gyrus were mainly observed in younger patients, whereas the bilateral caudate showed a main effect of psychiatric manifestations across age groups. This pattern suggests that the structural correlates of psychiatric manifestations in WD may involve both relatively stable subcortical regions and age-related cortical differences. However, our cross-sectional data cannot determine how these regional changes develop over time or whether they represent changes within the same neural circuit. Longitudinal studies combining structural and functional MRI are needed to clarify these relationships.

### 4.4 Possible mechanisms of psychiatric manifestations in WD

Wilson disease is caused by dysfunction of ATP7B, leading to impaired copper transport and abnormal copper accumulation in the liver, brain, and other tissues. Excessive copper in the central nervous system may contribute to oxidative stress, mitochondrial dysfunction, membrane damage, and neuronal injury. These processes may provide a biological basis for both neurological and psychiatric manifestations of WD (28).

The mechanisms underlying psychiatric manifestations in WD are likely to be complex. In addition to direct copper toxicity, changes in neurotransmitter systems may also be involved. Dopaminergic, serotonergic, and noradrenergic pathways are closely related to mood, motivation, cognition, and behavioral regulation. Disturbance of these systems may therefore contribute to the heterogeneous psychiatric manifestations observed in WD. Oxidative stress, mitochondrial dysfunction, neuroinflammation, and immune dysregulation may also participate in this process (28, 29). However, current evidence does not support a single mechanism that can fully explain psychiatric manifestations in all patients with WD.

The clinical findings of the present study provide additional context for these possible mechanisms. Patients with psychiatric manifestations had lower serum ceruloplasmin levels and a higher proportion of positive family history than those without psychiatric manifestations. Lower ceruloplasmin may reflect greater disturbance of copper metabolism, which is consistent with the central role of abnormal copper handling in WD. However, serum ceruloplasmin does not directly reflect copper accumulation in the brain, and the observed difference should not be interpreted as evidence that lower ceruloplasmin causes psychiatric manifestations. Similarly, the difference in family history was a descriptive finding and should not be considered an independent risk factor.

Taken together, psychiatric manifestations in WD may result from the combined effects of abnormal copper metabolism, cellular injury, altered neurotransmission, and other disease-related processes. Further studies combining clinical, biochemical, genetic, and multimodal imaging data are needed to clarify the mechanisms linking copper metabolism to psychiatric manifestations in WD.

## 5 Conclusions and limitations

In conclusion, psychiatric manifestations in WD were associated with regional gray matter differences. Significant age × psychiatric-manifestation interactions were observed in the left insula/Rolandic operculum and right inferior temporal gyrus, with lower GMV mainly observed in younger patients with psychiatric manifestations. Patients with psychiatric manifestations also showed lower GMV in the bilateral caudate nuclei. Further analyses showed that lower caudate GMV was associated with higher UWDRS-PSY scores. These findings suggest that age and caudate structure may be relevant to psychiatric manifestations in WD.

Several limitations should be acknowledged. First, the cross-sectional design precludes inference about temporality or causality between psychiatric involvement and gray-matter alterations. Second, no structured psychiatric diagnostic interview was performed. Psychiatric manifestations were assessed using the UWDRS psychiatric subscale and a binary grouping strategy, which may not fully capture the heterogeneity of psychiatric symptoms. Third, age was divided using a 30-year cutoff. Although sensitivity analyses using different age cutoffs showed similar results, age dichotomization may still result in some loss of information. Fourth, the number of older patients without psychiatric manifestations was relatively small, which may have reduced the ability to detect group differences. Fifth, detailed information on anti-copper treatment duration and medication exposure was incomplete and therefore could not be included as covariates. Residual confounding related to chelation or zinc treatment cannot be excluded. Finally, the caudate clusters used in the correlation analyses were identified from the psychiatric-manifestation comparison in the same sample. Therefore, these correlations were not statistically independent and should be interpreted as exploratory.

## Data Availability

The raw data supporting the conclusions of this article will be made available by the authors, without undue reservation.
